# Bioactive Molecules Delivery through Ferritin Nanoparticles: Sum Up of Current Loading Methods

**DOI:** 10.3390/molecules29174045

**Published:** 2024-08-27

**Authors:** Rosanna Lucignano, Giarita Ferraro

**Affiliations:** Department of Chemical Sciences, University of Naples Federico II, Complesso Universitario di Monte Sant’Angelo, via Cinthia, 26, 80126 Naples, Italy; rosanna.lucignano@unina.it

**Keywords:** ferritin nanocages, bioactive molecules, encapsulation, loading protocols, drug delivery

## Abstract

Ferritin (Ft) is a protein with a peculiar three-dimensional architecture. It is characterized by a hollow cage structure and is responsible for iron storage and detoxification in almost all living organisms. It has attracted the interest of the scientific community thanks to its appealing features, such as its nano size, thermal and pH stability, ease of functionalization, and low cost for large-scale production. Together with high storage capacity, these properties qualify Ft as a promising nanocarrier for the development of delivery systems for numerous types of biologically active molecules. In this paper, we introduce the basic structural and functional aspects of the protein, and summarize the methods employed to load bioactive molecules within the ferritin nanocage.

## 1. Ferritin: Structure and Biological Functions

Ferritin (Ft) is ubiquitous in all forms of cellular life, with the exception of yeast [1], and has wide-ranging functions. It is present in many cell types in humans and other vertebrates, in invertebrates, higher plants, fungi, and bacteria. Ferritin has been characterized as a cytosolic protein, but it is also present in other cell compartments, such as the nucleus, mitochondria, and lysosomes; a small amount of ferritin is located in the serum [2]. The role of ferritin in different cell types includes specialized functions, such as recycling iron in macrophages, short- and long-term iron storage in the red cells of embryos or hepatocytes of adults, and intracellular housekeeping functions, like providing reserves of iron for cytochrome, nitrogenase, ribonucleotide reductase, hemoglobin, myoglobin, and detoxification, if an excess of iron is present in the cell [3].

Ferritin is a multimeric protein ca 12.0 nm in diameter and ca 1.0 nm thick, with a mass of ca 450 kDa, which forms a nanocage structure that can contain thousands of iron atoms in the form of hydrous ferric oxide [Fe(III)O·OH] [1,2,4,5]. It has been reported that ferritins can be loaded with up to 3000 iron atoms, even if the correct amount is hard to determine due to iron binding to the Ft external surface, which decreases protein solubility [6]. The minerals have various amounts of phosphate (P) that reflect local phosphate concentrations; phosphate is low in animal ferritin iron minerals (Fe:P = 8:1) and high in plants or microorganisms (Fe:P = 1:1), where the ferritin minerals are largely amorphous.

**Figure 1:**
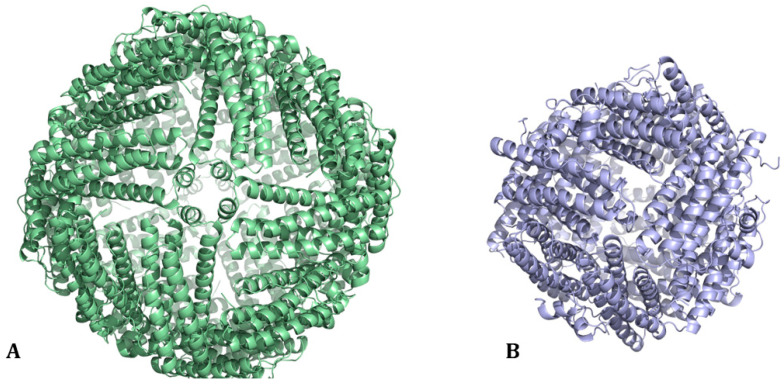
(**A**) The 24-mer cage structure of classical ferritin (Ft) and of bacterioferritin (Bfr from Escherichia coli, PDB code: 1BFR [1]). (**B**) The 12-mer cage structure of Dps from Escherichia coli (PDB code: 1DPS [2]).

The ferritin protein superfamily can be divided into three major subclasses: classical ferritins (Fts), heme-containing bacterioferritins (Bfrs), and DNA-binding proteins from starved cells (Dps). Ft and Bfr are made of 24 subunits (Figure 1A), whereas Dps are smaller with 12 subunits (Figure 1B). Ft is found in plants, bacteria, and animals, while Bfr and Dps are restricted to prokaryotes. A phylogenetic network analysis based on structure and sequence similarity concluded that all three subclasses share a common ancestor [7].

**Figure 2:**
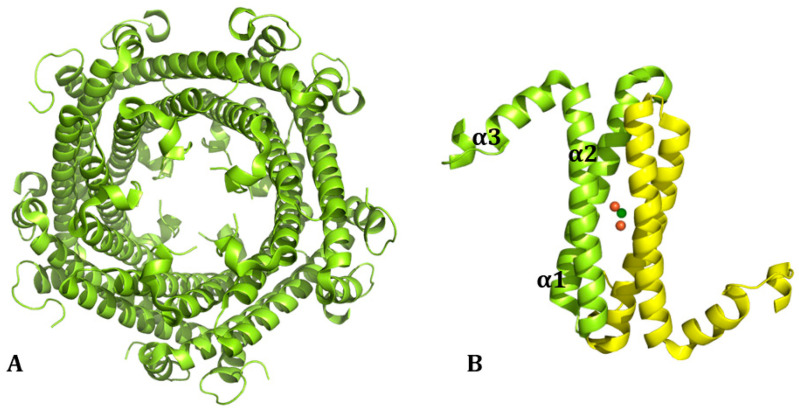
(**A**) Structure organization of the cage structure of ncapsulated ferritin from *Rhodospirillum rubrum* (EncFt; PDB code: 5DA5 [4]). (**B**) The single chain dimer is also reported.

In addition, there is a newly found form of ferritin, the encapsulated ferritin, recently discovered in bacteria and archaea [4], whose assembly pathway has been characterized by mass spectrometry [8]. This encapsulated ferritin (EncFt) has two main alpha helices (α1 and α2), which assemble in a metal-dependent manner to form a ferroxidase center at a dimer interface and a smaller helix (α3). EncFt adopts an open decameric structure that is topologically distinct from other ferritins (Figure 2). The doughnut-like decamer is 7.0 nm in diameter and 4.5 nm thick.

While EncFt acts as a ferroxidase, it cannot mineralize iron [9]; conversely, the encapsulin shell associates with iron, but is not enzymatically active [10]. It has been demonstrated that EncFt must be housed within the encapsulin for iron storage [8]. This encapsulin nano-compartment is widely distributed in bacteria and archaea, and represents a distinct class of iron storage system, where the oxidation and mineralization of iron are distributed between two proteins.

**Figure 3:**
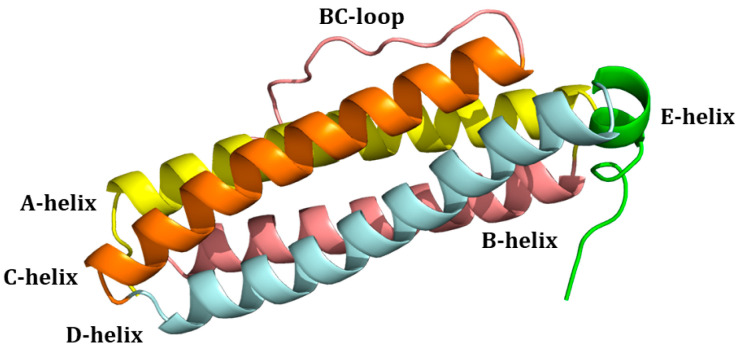
Structure of the four-helix-bundle of the ferritin subunit. Each subunit and the connecting loops are indicated.

Mammalian ferritins are composed of two types of chains: heavy (HFt) and light (LFt). HFt contains 178 amino acids with a molecular weight of 21 kDa, while there are 174 residues in LFt, which has a molecular weight of 19 kDa. Initially called H and L for their initial separation from the heart and liver [7], respectively, they share a common 3D structure [11]. Indeed, each subunit is folded into four long α-helices denoted as A, B, C, and D. The four long helices are arranged in an anti-parallel manner into a spiral bundle. The B-helix is connected to the C-helix by a B-C-loop consisting of 18 amino acids. There is also a long loop between the C- and D-helices. A short helix (E) is located at the end of the helix cluster. Helix E forms an angle of 60° with the B-C helices (Figure 3). Interestingly, there are differences between the hydrogen bonds and salt-bridges in the residue side chains of HFt and LFt. HFt has 50% more hydrogen bonds than LFt. Furthermore, there is an iron-binding site in the α-helical bundle of HFt, which has ferroxidase activity. In the LFt, the salt bridge between Lys62 and Glu107 replaces the iron-binding site of HFt. This interaction stabilizes the structure, which makes ferritin highly stable at high temperatures and in the presence of chemical denaturants [12].

**Figure 4:**
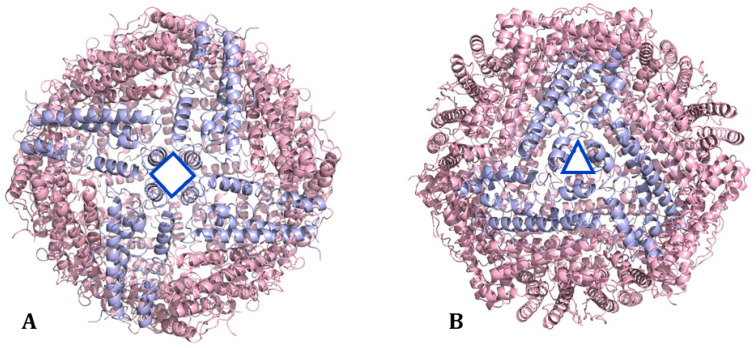
Organization of ferritin subunits to form (**A**) the four-fold channel C4 and (**B**) the three-fold channel C3 (PDB code: 5N27) [19].

HFt/LFt ratio varies in different organs [13,14]. The liver and spleen, mostly involved in iron storage, contain up to 90% of LFt, which is more stable and stores more iron than HFt. The heart and brain, implicated in high iron oxidation, contain mainly HFt, which has significant antioxidant activity [15]. Ferritin nanocages present two kinds of channels: four-fold hydrophobic channels (C4) and three-fold hydrophilic channels (C3) [16] (Figure 4). Four-fold hydrophobic channels promote proton transfer in or out of ferritin to maintain electrical neutrality during iron deposition; iron quickly passes through the ferritin shell mainly through three-fold hydrophilic channels. The hydrophilic region of the three-fold channel is surrounded by a negative charge generating an electrostatic field, which facilitates the introduction of Fe^2+^ to the channel entrance. Cys130 and His118 at the external and internal openings of the channel combine with iron ions. Then, iron enters ferritin to form iron cores with a size of 3.5–7.5 nm [17,18].

## 2. Ferritin as Delivery System for Cargo Molecules

The discovery of drug nanocarriers is a noteworthy step for pharmacological therapies attempting to improve patients’ safety and life quality [20,21,22,23]. Drug nanocarriers are particles with a nanometer size range, which results in advantageous properties, including a large surface-to-volume ratio and rapid dispersion. The large surface-to-volume ratio permits the addition of modification sites in order to reach an adequate drug storage space. Because of rapid dispersion, drug nanocarriers can be readily used in formulations. In addition, drug nanocarriers improve efficacy and safety in disease therapy via pharmacokinetic profile improvements and targeted delivery. They function to prevent drugs from unwanted degradation and interactions, both in storage and in vivo. They are cleared from circulation relatively slowly when compared to molecules of other sizes. Moreover, some drug nanocarriers have controlled or sustained drug release at targeted areas [24].

Besides having these basic properties of protein-based nanoparticles, ferritin has other attractive characteristics, including thermal stability, with a melting temperature of up to 80 °C, stability in extreme pH conditions (pH 3–12), easy surface functionalization, and low cost for large-scale production [25,26]. In addition, ferritin has an intrinsic targeting ability as a drug nanocarrier. The human H-chain ferritin (hHFt) subunit has been shown to selectively bind to a specific human receptor. Li et al., by using expression cloning, identified the transferrin receptor 1 (TfR1) as an important receptor for hHFt with little or no binding to hLFt (human L-chain ferritin) [27]. After binding to TfR1, hHFt enters not only endosomes but also lysosomes, which facilitates the release of iron from ferritin [28]. TfR1 is overexpressed in a range of tumor cells and the blood–brain barrier [29,30,31], thus representing an important target for ferritin-based therapy. Mice L-chain ferritin and horse spleen ferritin (mostly composed of L-chain) have an innate affinity to the scavenger receptor class A member 5 (SCARA5) [32]. Conti et al. found that cancer stem cells (CSC) enriched tumorspheres from breast cancer cell lines, displaying an increased L-chain ferritin uptake capability compared to their monolayer counterparts, as a consequence of the upregulation of the L-chain ferritin receptor SCARA5. Turino et al. reported an increased capability in hydrophobic PLGA nanoparticles to target MCF-7 (a human breast cancer cell line) upon decoration with L-chain ferritin [33]. The increased uptake and toxicity were confirmed by those obtained on MDA-MB-231 cells (human breast adenocarcinoma), used as negative control due to their lower SCARA5 expression.

## 3. Bioactive Molecules Loading

The natural function and structure of ferritin have opened the possibility of using it in the design of nanocarriers for the transport and delivery of molecules with different activities, ranging from drugs to nutrients [34], photosensitizers [35] and molecules for optical imaging [36,37,38,39], catalysis [40], and photodynamic therapy [41]. Metal nanoclusters [42], metal nanoparticles [43], metal compounds [44,45], and even entire proteins [46] have been trapped within the cage. This opportunity has prompted the development of different protocols for cargo molecules loading within the ferritin nanocage.

**Figure 5:**
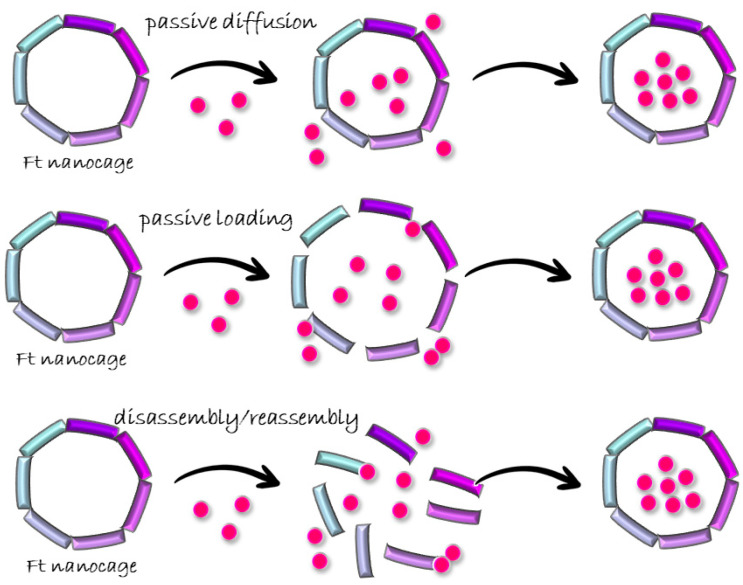
Scheme of ferritin cargo-loading approaches.

Drug loading comprises two steps: the entering of drugs into a cavity and the remaining of the drugs inside. Current drug-loading approaches use three distinct strategies in the first step: (1) biomineralization or passive diffusion, in which drugs diffuse inside the ferritin cavity through protein channels; (2) passive loading, which enlarges ferritin channels, allowing bigger molecules to enter; (3) disassembly–reassembly of the nanocages, which opens the ferritin cage to allow drug loading in the bulk and to restore the structure after encapsulating drugs [24] (Figure 5).

### 3.1. Passive Diffusion

The passive diffusion of cargo molecules through ferritin channels has been performed for small molecules. This process mimics the physiological iron biomineralization process. In fact, it is believed that the three-fold channel provides pathways for the transfer of ions. The transported ions bind a negatively charged metal-binding site in the inner cavity of the ferritin and forms a nucleus within the bulk. The nucleus grows into metal compound nanoparticles by free ion adsorption [47].

Inducible ferritins cloned from the oyster *Saccostrea cucullate* (rScFer) [48] and the hepatopancreas of the sea hare *Aplysia juliana* (rAjFer) [49] were loaded with CDDP (*cis*-diamminedichloroplatinum(II), cisplatin) through passive diffusion. The simple mixing of the proteins with CDDP, followed by overnight incubation, produced CDDP-rScFer/CDDP-rAjFer nanocomposites bearing 17.6 and 22.9 CDDP molecules per cage (detected by ICP-MS), respectively.

In the past, my research group obtained and characterized two different adducts upon the reaction of the recombinant hHFt with CDDP [19] and the antimetastatic ruthenium compound NAMI-A ([ImH][*trans*-RuCl_4_(dmso-S)(Im)]; dmso-S = sulfur-bonded dimethyl sulfoxide, Im = imidazole) [50] by passive diffusion in a solid state. Crystals of drug-free hHFt were soaked in a solution containing a molar excess of CDDP and NAMI-A, respectively. During incubation, the metal complexes pass through the solvent channels of the crystal to reach the protein bulk. The X-structure of the hHFt-CDDP adduct revealed that cisplatin directly interacts with the protein upon binding of the side chains of His105, His136, Lys68, Cys90, and Cys102 [19]. ICP-OES detected from 32 up to 47 Pt atoms per cage. The presence of CDDP did not alter the overall conformation of the protein, but completely abolished the ferroxidase activity of hHFt, probably due to competition at the level of the ferroxidase site. In the case of the adduct with NAMI-A, ruthenium ions bound the side chain of His105 [50]. The ruthenated ferritin had a slightly lower ferroxidase activity compared to the native form, not completely suppressed since Ru is bound far from the catalytic site.

The passive-diffusion approach has also been revealed to be particularly successful for the polymerization of molecular complexes within the nanocage upon the diffusion of reagents into ferritin channels. In this way, the nanocage provides a platform not only for the storage but, also, for the growth of various materials, including metal complexes, scaffolds for catalytic reactions, and nanoparticles.

**Figure 6:**
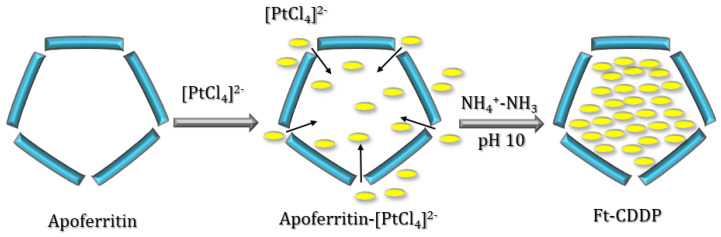
Scheme of the in situ generation of Ft-CDDP.

Yang and et al. took advantage of molecules diffusion towards ferritin channels to prepare ferritin-CDDP nanocomposites by an in situ procedure, using horse spleen ferritin (apo form, apoFt) [51]. An excess of K_2_PtCl_4_ (500: 1, Pt/Ft cage) was slowly added to a ferritin solution at pH 8.5. Then, apoFt-CDDP was obtained after the addition of NH_4_^+^–NH_3_ buffer solution at pH 10 to the AFt-PtCl_4_ solution (Figure 6).

The entrapment of PtCl_4_ in apoFt was confirmed by transmission electron microscopy (TEM), where reduced discrete spherical Pt particles with a relatively homogeneous size of 2–3 nm were observed, and by UV-vis spectroscopy. Indeed, the UV-vis spectrum of the apoFt-Pt solution showed a shoulder-band at 280 nm due to the encapsulation of Pt particles, which were formed by the reduction of PtCl_4_.

**Figure 7:**
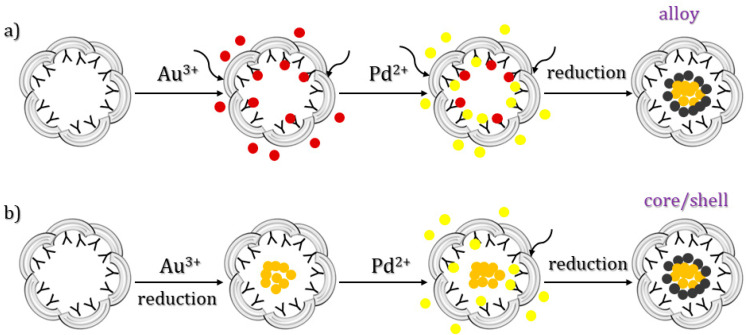
Schematic drawings of the methods of preparation of Au/Pd bimetallic NPs in apoLFt. Au^3+^, Au^0^, Pd^2+^, and Pd^0^ atoms are colored red, orange, yellow, and brown, respectively. In scheme (**a**) the reduction occurs upon loading of both gold and palladium; in scheme (**b**) the two metals are reduced each one upon loading.

Suzuki et al. prepared catalytic Au/Pd bimetallic nanoparticles within recombinant L-chain apo-Ft, from horse liver, by the co-reduction of a mixture of Au^3+^ and Pd^2+^ ions in apoLFt, thus improving the catalytic reactivity of olefin hydrogenation relative to Pd^0^ nanoparticles in the cage [52] (Figure 7). The nanoparticles were grown inside the cage upon the sequential incubation of apoferritin with KAuCl_4_ and KPdCl_4_ at pH 8.5. They took advantage of the different binding sites of Au and Pd on ferritin, unveiled by X-ray crystallography, and let metal ions diffuse and form clusters close to different protein residues. The formation of nanoparticles was then induced by metal reduction upon titration with NaBH_4_.

### 3.2. Passive Doading

#### Organic Solvents

**Figure 8:**
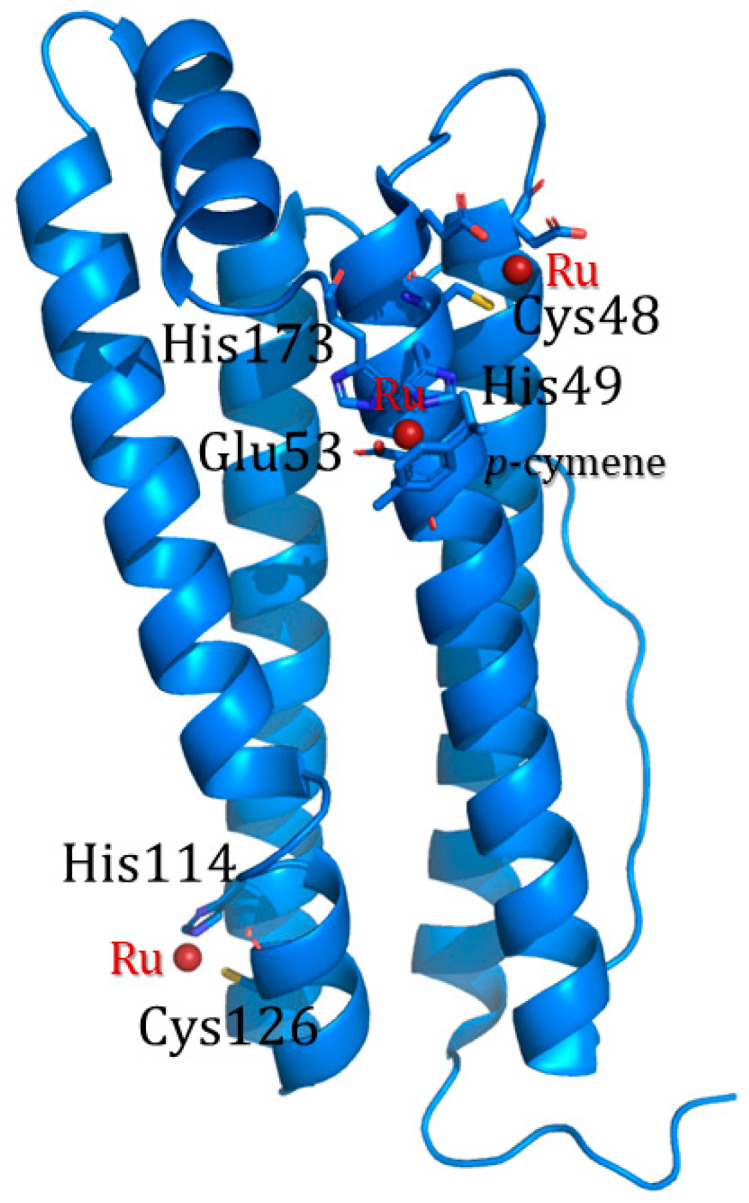
Subunit structure of Ru(*p*-cymene)-apoLFt. Ruthenium binding sites are shown as sticks and ruthenium atoms as dark red spheres [53].

The use of organic solvents induces a relaxation of the ferritin cage structure, favoring the entrance of the cargo molecules through the enlarged channels. Different solvents can be chosen for this scope. The Watanabe research group loaded three different mono-substituted ferrocene complexes into horse liver L-apo-ferritin by simply mixing ferritin and the iron complex in acetonitrile [53]. Incubation was followed by overnight dialysis against 0.15 M NaCl to remove unbound ferrocene molecules. The successful encapsulation was confirmed by ICP-MS. Takezawa et al. used the same approach to produce ferritin-based nanocomposites loaded with a ruthenium half-sandwich compound, Ru(II)(η^6^-*p*-cymene) (*p*-cymene = 1-methyl-4-(1-methyletheyl)benzene) [54] and rhodium complex [Rh(nbd)Cl]_2_ (nbd = norbornadiene) [55]. Regarding Ru(*p*-cymene)-apoLFt, ICP-OES data indicated the presence of about 88 atoms of Ru per cage, while X-ray crystallography allowed the identification of 72 Ru-binding sites per cage (three binding sites per single subunit). In particular, the X-ray structure analysis indicated the location of Ru complexes at the three-fold axis channel of the protein, where a Ru atom coordinates His114 and Cys126, and close to a metal-binding domain called an accumulation center. At the accumulation center, a Ru-*p*-cymene fragment interacts with His49, Glu53, and His173 on the interior protein surface, and a naked Ru ion coordinates a Cys48 side chain (Figure 8).

An identical result was obtained for the passive diffusion of the above-mentioned Rh compound and for a Pd-based complex, [Pd(allyl)Cl]_2_ (allyl = η^3^-C_3_H_5_) [56],within the same ferritin.

These findings indicate that the passive-diffusion method favors the accumulation of cargo molecules close to ferritin channels. Regarding Rh(nbd)-apoFt, this Rh-loaded nanocomposite was used as a scaffold for the polymerization of polyphenylacetylene, taking advantage of the catalytic activity of the encapsulated rhodium compound [55].

**Figure 9:**
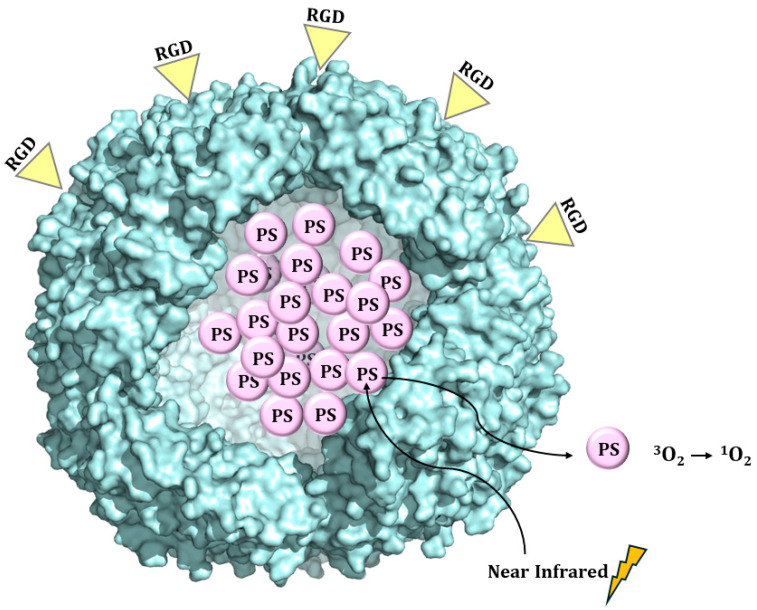
Schematic illustration of the formation and working mechanism of ZnF_16_Pc-loaded RFRT.

Targeted delivery is an important goal of photodynamic therapy, aimed at increasing tumor selectivity and the tumor/normal tissue accumulation ratio and avoiding off-target damage to the normal organs and surrounding tissues [35]. Zhen et al. loaded a RGD4C-modified ferritin (RFRT) with zinc hexadecafluorophthalocyanine (ZnF_16_Pc), a potent photosensitizer (PS) with a high ^1^O_2_ quantum yield and poor water solubility [35] (Figure 9). Drug loading was achieved by adding ZnF_16_Pc in DMSO into a RFRT solution in PBS pH 7.4 and, after that, incubating at room temperature for 45 min.

ZnF_16_Pc-loaded RFRT, tested on U87MG subcutaneous tumor models, showed high tumor accumulation rate, good tumor inhibition, and minimal toxicity to the skin and other major organs.

An overnight incubation at 4 °C in a 1:1 DMSO/PBS solution under stirring prompted the loading of Gefitinib, an orally active, epidermal growth factor receptor (EGFR) tyrosine kinase (HR2) inhibitor that is part of breast cancer (SKBR3) therapy [57] within human H-chain ferritin [58]. Gefitinib-loaded ferritin showed increased cellular uptake, and potent and enhanced antitumor activity against the HER2 overexpressing SKBR3 cell line compared to Gefitinib alone, making it a promising carrier for the delivery of drugs to tumor sites.

### 3.3. Disassembly/Reassembly

#### 3.3.1. pH Switch Disassembly/Reassembly Protocol

Ferritin stability in a wide range of pHs was exploited to set up an encapsulation protocol based on the pH switch. Kim et al. closely analyzed Ft cage architecture in response to different pHs by SAXS [59]. They showed that, under neutral conditions, Ft assumes a hollow spherical structure identical to that determined in the crystalline state, and that the spherical cage remains stable over the pH range 3.4–10. Below pH 3.4, ferritin becomes unstable and undergoes stepwise disassembly through several structural intermediates: a hollow spherical structure with two holes, a headset-shaped structure, and, ultimately, rodlike oligomers (mainly trimers) or monomers. Below pH 0.80, the disassembled subunits undergoes aggregation, which was attributed to denaturation. Structural recovery is then achieved by the restoration of pH to a neutral value [59].

Based on these findings, encapsulation procedures based on the disassembly at acidic (pH = 2) or even at alkaline pHs (pH = 13) have been employed to load different types of molecules within the cage.

Disassembly at acidic pH was described for the first time in 1994 by Webb et al. [60] as he tried to entrap a series of small molecules with different chemical natures within horse spleen ferritin: an iron chelator (2,2′-bipyridine), the redox active molecules methyl viologen and flavin mononucleotide, and two pH indicators: neutral red and phenolphthalein [60]. By using capillary electrophoresis, they demonstrated the association of small molecules to Ft after the pH-induced unfolding/refolding process. Since then, this procedure has been the preferred method for obtaining nanocomposites with encapsulated cargo molecules.

The acidic pH switch disassembly/reassembly protocol was chosen by Simsek et al. to produce doxorubicin(DOX)-encapsulated apoferritins [61]. They disassembled an apoferritin solution into a 20 mM glycine-acetate buffer at pH 2.5 and incubated the solution with DOX at different concentrations. They slowly raised the pH to 4 by adding 4.0 M trizma-base solution and performed overnight dialysis against 20 mM trizma-base-acetate buffer at pH 7.4, which neutralized the pH. A final wash in phosphate buffer at pH 7.4 removed the unbound doxorubicin. The encapsulation efficiency was very low. The highest drug encapsulation was found at a ratio of 1/5 (ferritin cage/DOX) and was accompanied by a huge protein loss upon acidic treatment, from 36 to 68% (Table 1).

Many metal-based compounds of medicinal interest have been encapsulated by this method, including the anticancer drugs cisplatin and carboplatin (*cis*-diamine(1,1-ciclobutandicarbossilate) platinum(II)) [51] and hydrophobic ruthenium(II)polyridyl complexes Ru(bpy)_2_dppz^2+^ and Ru(phen)_2_dppz^2+^ [62].

**Figure 10:**
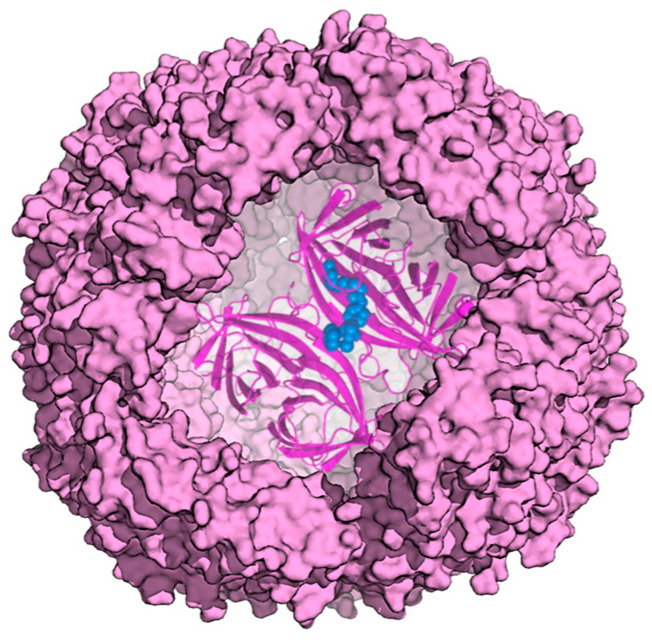
Model of ATHase within apoferritin. Enzyme cofactor is depicted as blue spheres.

A ferritin hollow cage structure can also offer an ideal capsule to host a protein. In this respect, an artificial metalloenzyme, a transfer hydrogenase (ATHase), was compartmentalized within horse spleen ferritin to exploit the reversible dissociation and reassembly of apoferritin at pH 2.0 [46] (Figure 10).

Biotin-streptavidin technology was employed to provide a well-defined second coordination sphere around the metal moiety and to ensure cofactor localization. The ferritin shell provided a third coordination sphere, which significantly influenced ATHase catalytic activity for the reduction of cyclic imines. Indeed, the resulting ATHases maintained their catalytic activity, displaying up to >3800 TON for the reduction of cyclic imines. Upon encapsulation, the ATHase showed a marked variation in enantioselectivity, resulting from the combined effect of both the second and third coordination sphere provided by Sav and ferritin, respectively.

**Figure 11:**
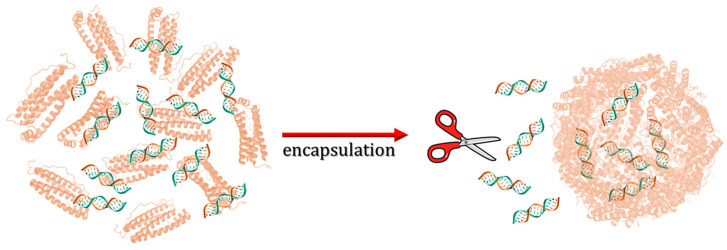
Schematic illustration of siRNA loading within human H-chain ferritin.

Speaking of encapsulating huge molecules within ferritin nanocages, Li et al. developed ferritin nanocarriers for the delivery of small interfering RNA (siRNA) [63]. RNA loading was performed by disassembling hHFt at pH 2 according to the standard procedure discussed above (Figure 11). The treatment of siRNA-Fts with RNases removed siRNA molecules on the ferritin outer surface.

Compared to lipofectamine, commonly used for the transfection of primary and tumorigenic cells [64], as an endogenous delivery agent, apoferritin does not induce the immune activation of T- and B-cells in human PBMCs (peripheral blood mononuclear cells). The presence of a ferritin shell confirmed highly efficient siRNA delivery in human primary and tumor cells. Moreover, the silencing effects of siRNA-hHFt outperformed those with lipofectamine in the evaluated concentration range.

**Figure 12:**
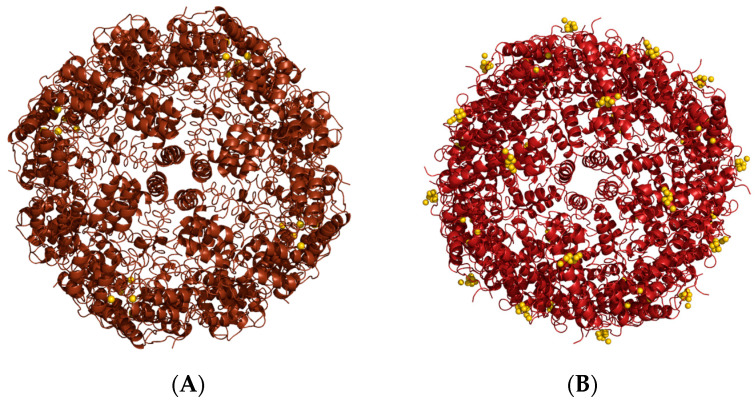
Cartoon representation of the cage structures of (**A**) Auranofin-loaded horse spleen ferritin and (**B**) Auranofin-loaded human H-chain ferritin. Gold atoms are depicted as yellow spheres.

Horse spleen ferritin has been used to encapsulate several metallodrugs [65,66,67,68,69,70] by dissociation at alkaline pH, from cisplatin [65] and carboplatin [71] to gold [66,70,72], ruthenium [67], and other platinum-based metallodrugs, including homo-bimetallic [66] and hetero-bimetallic [68,69]. The nanocomposites have been characterized by X-ray crystallography. This technique allowed us to evaluate the efficiency of the encapsulation protocols and to identify the number and location of metal-binding sites. The drug-loaded nanocomposites, mostly prepared by our research group, showed a high loading efficiency, dependent upon the nature of the encapsulated metallodrugs. It has been reported that a ferritin nanocage can encapsulate up to 600–700 metal atoms [66,69]. A summary of the results obtained using this approach has been reported elsewhere [73]; here only a few examples are recalled. It is worth mentioning a comparative study of the encapsulation abilities of horse spleen ferritin and recombinant human H-chain ferritin upon alkaline disassembly [72]. Auranofin (AF), an anti-arthritic Au(I) compound already used in clinical practice [74], was encapsulated in both ferritins, giving a different result, unexpectedly. Indeed, the two nanocomposites presented significant differences in the distribution of gold-binding sites. In the nanocomposite with horse spleen ferritin, most of the gold atoms detected by ICP-AES (270 Au atoms/cage) were trapped in the bulk, since the X-ray structure indicated one gold-binding site with partial occupancy close to Cys126, a residue buried at the interphase between two subunits (Figure 12A). In the nanocomposite with human H-chain ferritin, a lower number of gold atoms were detected (90 Au atoms/cage), and many were localized on the protein surface (Figure 12B).

These differences indicated the different responses of the two ferritins to the method selected for the disassembly, suggesting that the experimental conditions for Ft cages dissociation can influence the behavior, not only of the cargo molecules but also of the carrier itself.

Conversely, Conti et al. reported on the successful encapsulation into hHFt of two highly charged Ru(II) polypyridyl complexes by the disassembly of the protein at pH 12 [75]. The resulting Ru(II)-ferritin nanocomposites were highly luminescent and displayed great stability in physiological conditions. The Ru compounds maintained their singlet oxygen-sensitizing properties and acquired cell selectivity towards HeLa and A2780 cells, overexpressing TfR-1, once encapsulated within hHFt [27].

hHFt was used to encapsulate β-carotene [76] at pH 11. β-carotene is a typical compound among carotenoids, which has a number of potential health benefits for human. Its employment is limited mostly due to poor water solubility and low thermal stability [77]. High-performance liquid chromatography (HPLC), UV-Vis spectroscopy, and TEM indicated that β-carotene molecules were successfully encapsulated within protein cages with a β-carotene/protein molar ratio of 12.4/1. These β-carotene-containing apoferritin nanocomposites were water-soluble. Interestingly, the thermal stability of the β-carotene encapsulated within apoferritin nanocages was markedly improved when compared to free β-carotene.

#### 3.3.2. Use of Chemical Denaturants

Chemical agents have also been used to disassemble and reassemble ferritin nanocages. In particular, protocols based on the use of urea and SDS have been developed.

**Figure 13:**
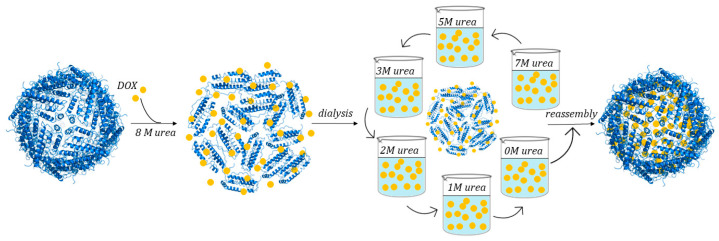
Schematic illustration of DOX loading within human H-chain ferritin by urea-based protocol.

hHFt-DOX nanoparticles were prepared by loading DOX into the cavities of hHFt nanocages through disassembling in 8 M urea, in the presence of DOX, followed by a reassembling process with a series of stepwise gradients of urea from 8 M to 0 M in PBS buffer [78] (Figure 13).

Cyanin5.5-labeled hHFt-DOX, specifically bound and subsequently entered HT29 tumor cells (colon adenocarcinoma) via interaction with overexpressed transferrin receptor 1, released DOX into the lysosomes. In vivo hHFt-DOX exhibited intra-tumoral drug concentration more than 10 times higher than that of free DOX, and significantly inhibited tumor growth after a single-dose injection. Importantly, hHFt-DOX displayed an excellent safety profile that significantly reduced healthy organ drug exposure and improved the maximum tolerated dose by four times compared with free DOX.

Most of the drug-loaded nanocomposites prepared by the pH switch disassembly/reassembly protocol reported in the literature predominantly use horse spleen ferritin and only rarely use the human variant. The above-discussed encapsulation of the same Au(I) complex within horse spleen ferritin and hHFt, following the same protocol [72], showed significant differences, especially regarding the loading efficiency and localization of gold on the inner or outer surface of the protein cage, demonstrating the inefficiency of this encapsulation protocol for hHFt. Most of the gold compound, in high molar excess to the respect of the protein, remained out of the cage. By investigating the mechanisms at the basis of the disassembly/reassembly processes of hHFt, our research group revealed how the alkaline treatment does not allow hHFt nanocages to disassemble, and how the acid treatment prompts hHFt disassembly, though not completely [79]. For this reason, we developed a new encapsulation procedure that uses small concentrations of the denaturing agent SDS (sodium dodecyl sulphate) to disassemble the nanocage. A percentage of 0.1% is enough to disassemble the cage. The reassembly occurs upon SDS removal through extensive dialysis. The SDS-based protocol proof was obtained by encapsulating two types of molecules: a ruthenium compound and a hemolytic peptide. Successful ruthenium encapsulation was verified by a combination of X-ray crystallography and UV-vis spectroscopy. In addition, ICP-MS confirmed the loading of about 10 ruthenium atoms per cage. The presence of the peptide within ferritin was confirmed thanks to a coupled approach combining gel electrophoresis under denaturing conditions and MALDI mass spectrometry [80]. An average of one peptide molecule per cage was encapsulated.

### 3.4. One-Step Method

Inoue et al. established a simple high-yield process for preparing high-drug-loaded ferritin for industrial production in a one-step method [80]. In particular, the drug-loaded ferritin was prepared under mild experimental conditions. Human H-chain ferritin in Tris-HCl buffer at pH 9 was incubated with DOX for 1 h at 60 °C. Unbound DOX was removed by ultrafiltration. To evaluate the efficiency of this new procedure, they prepared the same nanocomposite by the disassembly/reassembly of ferritin at acidic pH. Compared to the disassembly/reassembly method, the loading capacity of a DOX-loaded hHFt, constructed by this novel method, was over 3 times higher, while DOX recovery was 10 times higher. A characterization by TEM, size exclusion chromatography, dynamic light scattering, and zeta potential have indicated that DOX-hHFt exhibits the same physicochemical features as natural apo-ferritin. Moreover, DOX-hHFt can be taken up to induce the apoptosis of colon26 cells (a mouse cell line derived from colon cancer) that are overexpressing TfR1.

Performing drug encapsulation by this method, the authors hypothesized that DOX might be encapsulated by the same mechanism used in nature by ferritin for iron loading. However, the diameters of metal ions are in the range of a few angstroms and are smaller than the size of DOX, which is over 0.94 nm. They took into account the fact that the four-fold channel is 0.9 nm in diameter [11] and could be expanded to more than 1.0 nm via thermal fluctuation. Moreover, the electrostatic charge is also expected to facilitate the passage of organic molecules through the pores traversing the ferritin shell. According to the Nernst equation, the loading capacity of a drug also depends on the charge of both the ferritin and the drug. Indeed, at a pH above 5, the surface and inner cavity of ferritin are negatively charged, and the surface potential of ferritin is slightly negative from pH 6 to 9. Since the pKa of DOX is 8.2, as the pH decreases, the proportion of the positively charged DOX molecules increases and predominates; therefore, the optimal value of the DOX-encapsulation reaction pH should be around 6. Indeed, the loading capacity at pH 6 was two times higher compared to that at pH 5; however, the most efficient encapsulation was observed in the pH 9 solution, indicating that the encapsulation reaction of DOX was not a simple reaction dependent upon electrostatic charge potential. The one-step encapsulation reaction therefore comprises two processes: the first involves the loading of a drug molecule, driven by the electrostatic charge potential; the second involves the deposition of the drug molecule in the ferritin inner cavity. Although the number of positively charged DOX molecules is small at pH 9, the strong potential difference between the ferritin inner surface and the shell may be sufficient to transport DOX molecules. Moreover, to increase the loading capacity, it is important to select a pH near the pKa in order to promote the deposition of the molecule in the ferritin cavity. To confirm this theory, Inoue et al. encapsulated different organic small molecules by the one-step method [80]. They set up optimal reaction conditions for each molecule, and they found that drug-loaded nanocomposites based on ferritin can be obtained by the reaction at pH near the pKa of target molecules.

### 3.5. PINCs Method

**Figure 14:**
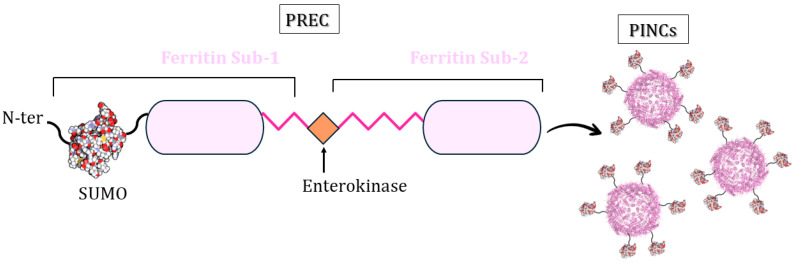
Schematic representation of PREC design: two subunits of *Pyrococcus furiosus* ferritin (PfFtn) are linked by a peptide with an enterokinase cleavage site and a SUMO protein linked to the N-terminus of Sub-1.

Sheng et al. described a new loading method that uses a modified ferritin [81]. They developed an engineering approach mimicking the HIV-1 (Human Immunodeficiency Virus 1) virion maturation process [82,83] to control ferritin nanocage assembly. Inspired by the HIV-1 Gag polyprotein precursor, multiple PREcursors of nanoCage (PREC) were created using 24-mer ferritin subunits, attaching the C-terminal of one ferritin subunit (Sub-1) to the N-terminus of a second (Sub-2) using a linker peptide with an enterokinase cleavage site (Figure 14). They demonstrated that the protease cleavage of these precursors leads to spontaneous self-assembly and the formation of ferritin-like nanocages, named protease-induced nanocages (PINCs), without protein aggregation/precipitation. These precursors can have additional proteins at their N-termini. The authors chose to link the sequence of a SUMO protein (Small Ubiquitin-like Modifier) to the N-terminus of Sub-1 as a proof-of-concept (Figure 14).

To produce the new Ft, they started from the subunits of the hyperthermophilic ferritin from *Pyrococcus furiosus* (PfFtn) as a model. This choice was made because PfFtn has extreme thermostability (>100 °C) [84] and shows notable immunogenicity and biosafety as a drug delivery vehicle [85] or as a platform for making vaccines [86].

They showed how PINC formation allows not only concurrent surface decoration with a protein, but also hydrophilic or hydrophobic drug encapsulation up to four times more than the amount achieved using other methods. To do this, two different molecules were selected to be loaded within PINC ferritin: a hydrophilic one, the doxorubicin, and a hydrophobic one, the anticancer drug camptothecin (CPT). Each drug was mixed with precursors (PREC). The enterokinase was added to catalyze PINCs assembly and passively encapsulate DOX and CPT, respectively. The amount of each drug that was encapsulated through the PINC method was in the range of 350–400 molecules per cage, far above the amount reported for other loading methods (30–40 molecules per cage) [78,87,88,89].

## 4. Conclusions

Ft nanocages are an excellent vehicle for different types of molecules, ranging from ions to entire proteins, as Fts can encapsulate them in their interior cavity using different protocols. Ferritin can mediate the selective transport of cargo molecules towards specific cellular targets, taking advantage of its uptake from receptors overexpressed in different cancer cell lines [30,32].

Three different modes of molecule loading within ferritin bulk have been analyzed, citing various works as examples. Passive diffusion mimics the natural function of Ft, which is iron storage. It uses the three- and four-fold channels of ferritin for molecule loading. Due to the small dimensions of the channel entrances, this method fits for the encapsulation of molecules with a diameter no bigger than 0.9 nm, which is the size of the largest Ft channel (the four-fold channel). In passive loading, drug loading occurs following the same mechanism as passive diffusion; however, in this case, the employment of organic solvents, like acetonitrile or DMSO, facilitates loading through the relaxation of the cage structure, which causes an enlargement of the ferritin channels. This allows cargo molecules to enter within the bulk. The disassembly/reassembly protocol, guided by extreme pHs or denaturing agents, is the most widely utilized method for loading bioactive molecules within Ft nanocages. Using this method, there are no limits in the size of the cargo molecules. Indeed, it was selected to encapsulate huge molecules like siRNA or artificial enzymatic proteins. Each method presents some drawbacks, which restrict their usage for the development of nanoparticles for different biotechnological applications. Some of these limits were exploited studying the behavior of human H-chain ferritin at pH 2 and pH 13, and prompted the development of a new, improved protocol for disassembly. Compared to pH-switch-encapsulation methods, the SDS-based protocol demonstrated a series of advantages as it is able to overcome common encapsulation procedure drawbacks. Indeed, it showed quantitative protein recovery upon SDS treatment. A high percentage of protein loss is a huge limitation with respect to pH-switch-based protocols. Moreover, encapsulation under mild pH conditions does not restrict the kind of molecule to be encapsulated since it removes the pH-sensitivity variable. Similar results were observed by encapsulation through the one-step method, which reached increased drug loading with maximum protein recovery.

Altogether, the results this paper has summarized underline the keen interest of the scientific community in expanding what is already known about ferritin and in developing efficient protocols to load bioactive molecules within the ferritin cage.

## Figures and Tables

**Table 1:** Final apoferritin concentrations.

Samples	Initial Concentration (μM)	Final Concentration (μM)	Percentage Lost (%)
FR1	2.5	0.8	68
FR8	2.5	1.6	36
FR17	2.5	1.5	40
